# Supplementary material for: Content-rich biological network constructed by mining PubMed abstracts
Source: BMC Bioinformatics. 2004 Oct 8;5:147. doi: 10.1186/1471-2105-5-147 (PMC528731; doi:10.1186/1471-2105-5-147)
Supplement: Additional File 2 — The original results of the above study (non-essential files are deleted to keep the file size under the limit set by BMC bioinformatics). [file 1471-2105-5-147-S2.bz2 › chilibotAdditionalFile2/dip50/437ID9430724E10382/html/CCL17_CCR4.html]

 


 **CCL17** and **CCR4** 
  
Found 87 abstracts in PubMed, retrieved 50.  
 

 What does Google say? 
 PDF only 
| .edu only 

---

**Interactive relationship** (e.g. stimulation, inhibition, etc)

**Neutral relationship**- TARC  [ **CCL17** ]  interacts primarily with the  **CCR4**  receptor and to a lesser extent with the CCR8 receptor.  Ref: 12832759 Acta Crystallogr D Biol Crystallogr, 2003
- TARC  [ **CCL17** ]  interacts primarily with the  **CCR4**  receptor and to a lesser extent with the CCR8 receptor.  Ref: 12499558 Acta Crystallogr D Biol Crystallogr, 2003
- Since the ligand of the TARC  [ **CCL17** ]  and MDC is  **CCR4** , which is specifically expressed on Th2 type T cells, the production of these CC chemokines is likely to play important roles in the T cell and B cell interaction.  Ref: 12715916 J Med Dent Sci, 2003
- The CC chemokine thymus and activation regulated chemokine  [ **CCL17** ]  TARC  [ **CCL17** ]   **CCL17**  acts on CC chemokine receptor 4  [ **CCR4** ]   **CCR4** , which is known to be selectively expressed in Th2 cells.  Ref: 12354417 J Dermatol Sci, 2002
- Furthermore, it was found that  **CCR4**  mRNA was preferentially expressed in lesional skin of dogs with AD, together with the mRNA of thymus and activation regulated chemokine  [ **CCL17** ]  TARC  [ **CCL17** ] , which is a ligand for  **CCR4** .  Ref: 12459162 Vet Immunol Immunopathol, 2002
- CONCLUSION Our findings imply that TARC  [ **CCL17** ]  secreted in decidua mediates the infiltration of  **CCR4**  T cell migration into the fetomaternal interface, decidua, resulting in the maintenance of pregnancy.  Ref: 12322891 Am J Reprod Immunol, 2002
- The helper Th 2 cell attracting chemokines thymus and activation regulated chemokine  [ **CCL17** ]  TARC  [ **CCL17** ]  and macrophage derived chemokine MDC are ligands for the chemokine receptor  **CCR4** .  Ref: 12645858 J Clin Immunol, 2003
- IP10, MIG and TARC  [ **CCL17** ]  positive HD cases contained higher numbers of Th2 lymphocytes ST2 CCR3 or  **CCR4**  positive lymphocytes, compared to IP10, MIG and TARC  [ **CCL17** ]  negative HD cases p <.  Ref: 11920617 Int J Cancer, 2002
- The dominance of CCL22 over  **CCL17**  extends to other  **CCR4**  mediated phenomena such as receptor desensitization and internalization and correlates with the peculiar kinetics of  **CCR4**  engagement by the two ligands.  Ref: 12193695 J Immunol, 2002
- A Th2 chemokine, TARC  [ **CCL17** ] , produced by trophoblasts and endometrial gland cells, regulates the infiltration of  **CCR4**  T lymphocytes into human decidua at early pregnancy.  Ref: 12322891 Am J Reprod Immunol, 2002
- Thymus and activation regulated chemokine  [ **CCL17** ]  TARC  [ **CCL17** ]  is known as a functional ligand for CC chemokine receptor 4  [ **CCR4** ]   **CCR4** , which is selectively expressed on Th2 lymphocytes and induces selective migration of the cells to allergic lesions.  Ref: 11642275 J Vet Med Sci, 2001
- Thymus and activation regulated chemokine  [ **CCL17** ]  TARC  [ **CCL17** ]   **CCL17**  is a member of the CC chemokines and is a chemoattractant for CC chemokine receptor 4  [ **CCR4** ]   **CCR4**  and CC chemokine receptor 8 CCR8 expressing cells.  Ref: 12522366 J Am Acad Dermatol, 2003
- ... interactions of  **CCR4**  with MDC CCL22 or TARC  [ **CCL17** ]   **CCL17** , CCR8 with I 309 CCL1, and CRTH2 with prostaglandin D 2 play a critical role in the allergen induced recruitment of Th2 cells in the target tissues of allergic inflammation.  Ref: 12009564 Mol Immunol, 2002
- Type 2 T cells have been shown to be preferentially recruited by the chemokines eotaxin CCL11, macrophage derived chemokine MDC, CCL22, and thymus activation regulated chemokine  **CCL17**  through their interaction with CCR3 and  **CCR4** , respectively.  Ref: 12023397 J Immunol, 2002
- CC chemokine receptor 4  [ **CCR4** ]   **CCR4**  has been identified as a specific receptor for both thymus and activation regulated chemokine  [ **CCL17** ]  TARC  [ **CCL17** ]  and macrophage derived chemokine MDC, and is preferentially expressed on Th2 cells.  Ref: 12757454 Allergy, 2003
- Only the high  **CCR4**  levels characteristic of skin lymphocytes were associated with robust chemotactic and adhesive responses to TARC  [ **CCL17** ] , consistent with a selective role for  **CCR4**  in skin lymphocyte homing.  Ref: 11786428 Am J Pathol, 2002
- T cells expressing CLA and  **CCR4**  were also found at high levels in cutaneous T cell lymphoma lesions along with abundant expression of the two  **CCR4**  ligands TARC  [ **CCL17** ]   **CCL17**  and MDC CCL22.  Ref: 12485447 J Invest Dermatol, 2002
- In addition,  **CCR4** , which is characteristic Th2 helper phenotype, and its ligand thymus and activation regulated chemokine  [ **CCL17** ]  TARC  [ **CCL17** ] , were compared with CXCR3, as Th1 phenotype.  Ref: 12688353 Leuk Lymphoma, 2003
- Recently, thymus and activation regulated chemokine  [ **CCL17** ]  TARC  [ **CCL17** ]   **CCL17** , a CC chemokine, was identified as a selective chemoattractant for CC chemokine receptor 4  [ **CCR4** ]   **CCR4**  expressing cells.  Ref: 12588369 Br J Dermatol, 2003
- Thymus and activation regulated chemokine  [ **CCL17** ]  TARC  [ **CCL17** ]  is a member of CC chemokine and plays an essential role in recruitment of CC chemokine receptor 4  [ **CCR4** ]  positive Th2 cells to allergic lesion.  Ref: 12655128 J Vet Med Sci, 2003
- BACKGROUND A Th2 chemokine, thymus and activation regulated chemokine  [ **CCL17** ]  TARC  [ **CCL17** ]   **CCL17** , produced by keratinocytes, is implicated in the development of atopic dermatitis by recruiting CLA  **CCR4**  lymphocytes into lesional skin and its expression was induced by proinflammatory cytokines such as interferon gamma IFN gamma and tumor necrosis factor alpha TNF alpha.  Ref: 12615364 J Dermatol Sci, 2003
- Thymus and activation regulated chemokine  [ **CCL17** ]  TARC  [ **CCL17** ]   **CCL17**  is a lymphocyte directed CC chemokine, which plays a role in the recruitment of CC chemokine receptor 4  [ **CCR4** ]  positive T helper 2 Th2 cells.  Ref: 11956056 Am J Respir Crit Care Med, 2002
- Thymus and activation regulated chemokine  [ **CCL17** ]  TARC  [ **CCL17** ]   **CCL17**  and macrophage derived chemokine MDC CCL22 are a pair of CC chemokines known to selectively attract T h 2 type memory T cells via  **CCR4** .  Ref: 12096036 Int Immunol, 2002
- PROBLEM A chemokine receptor,  **CCR4**  preferentially expressed on type 2 helper T Th2 type cells, and its ligand, thymus and activation regulated chemokine  [ **CCL17** ]  TARC  [ **CCL17** ]  CCL play important roles in the recruitment of Th2 type cells.  Ref: 12322891 Am J Reprod Immunol, 2002
- Three chemokines in particular, CXCL12 SDF 1,  **CCL17**  TARC  [ **CCL17** ] , and CCL22 MDC, recently have been shown to be strong and rapid activators of platelet aggregation and adhesion after their binding to platelet CXCR4 or  **CCR4** , when acting in combination with low levels of primary agonists.  Ref: 12851650 Microcirculation, 2003
- Treg cells specifically express the chemokine receptors  **CCR4**  and CCR8 and represent a major subset of circulating CD4 T cells responding to the chemokines macrophage derived chemokine MDC CCL22, thymus and activation regulated chemokine  [ **CCL17** ]  TARC  [ **CCL17** ]   **CCL17** , I 309 CCL1, and to the virokine vMIP I ligands of  **CCR4**  and CCR8.  Ref: 11560999 J Exp Med, 2001

**Non-interactive relationship** (e.g. studied together, co-existance, homology, etc.)

- **CCL17**  is displayed by cutaneous but not intestinal venules, and is thought to trigger vascular arrest of circulating skin homing memory T cells, which uniformly express the TARC  [ **CCL17** ]  receptor CC chemokine receptor CCR 4  [ **CCR4** ] .  Ref: 11714760 J Exp Med, 2001
- Some reports suggest that  **CCR4**  and TARC  [ **CCL17** ]  are important for the pathogenesis of atopic dermatitis.  Ref: 12677260 Drug News Perspect< MedlineTA>Drug News Perspect, 2001
- In view of the potential importance of  **CCR4**  in skin homing of T cells, we investigated the expression pattern of  **CCR4**  and its ligands TARC  [ **CCL17** ]   **CCL17**  and MDC CCL22 in the peripheral blood and skin of patients with cutaneous T cell lymphoma, a putative malignancy of the skin homing T cells.  Ref: 12485447 J Invest Dermatol, 2002
- These data further substantiate the role of TARC  [ **CCL17** ]   **CCR4**  in the pathogenesis of AD.  Ref: 12598706 J Dermatol, 2003
- CONCLUSIONS Our data suggest that Th2 cells, which express  **CCR4**  and its ligands TARC  [ **CCL17** ]  and MDC, contribute to the pathogenesis of EP in the lung.  Ref: 12757454 Allergy, 2003
- Both thymus and activation regulated chemokine  [ **CCL17** ]  TARC  [ **CCL17** ]   **CCL17** , a ligand for CC chemokine receptor 4  [ **CCR4** ]  that is mainly expressed on Th2 type cells, and interferon induced protein of 10kDa IP 10 CXCL10, a ligand for CXC chemokine receptor 3 that is mainly expressed on Th1 type cells, are produced by keratinocytes after the stimulation with the primary cytokines such as tumor necrotic factor alpha TNF alpha and or interferon gamma IFN gamma.  Ref: 12441140 Cytokine, 2002
- Cells expressing the  **CCR4**  ligand TARC  [ **CCL17** ]  thymus and activation regulated chemokine  [ **CCL17** ]  were detected within infiltrated islets from prediabetic mice.  Ref: 12464673 J Clin Invest, 2002
- We have examined the in situ expression of a typical Th2 type chemokine, thymus and activation regulated chemokine  [ **CCL17** ]  TARC  [ **CCL17** ] , and its receptor  **CCR4**  using immunohistochemical techniques.  Ref: 12456591 Int Immunol, 2002
- The small number of  **CCR4**  positive reactive T cells in most cases studied argues against an important role of TARC  [ **CCL17** ]  expression in the evasion of antitumor responses.  Ref: 12181269 Mod Pathol, 2002
- Type 1 chemokines CXC chemokine ligand CXCL 9 and CXCL10 monokine induced by IFN gamma and IFN gamma inducible protein of 10 kDa CXCR3 ligands, and type 2 chemokines CC chemokine ligand CCL 11 eotaxin CCR3 ligand,  **CCL17**  thymus and activation regulated chemokine  [ **CCL17** ]   **CCR4**  ligand and CCL22 monocyte derived chemokine  **CCR4**  ligand were readily detectable in the plasma of most participants.  Ref: 12407016 Int Immunol, 2002
- The expression and distribution of TARC  [ **CCL17** ]  and  **CCR4**  mRNAs were detected with the in situ reverse transcription RT polymerase chain reaction PCR technique.  Ref: 12598706 J Dermatol, 2003
- Cells transfected with gpCCR4 migrated toward the human and murine orthologues of the  **CCR4**  ligands, macrophage derived chemokine and thymus and activation regulated chemokine  [ **CCL17** ] .  Ref: 11724798 J Biol Chem, 2002
- Recently, macrophage derived chemokine MDC CCL22, a CC chemokine, was identified as a selective chemoattractant for CC chemokine receptor 4  [ **CCR4** ]   **CCR4**  expressing cells, in addition to thymus and activation regulated chemokine  [ **CCL17** ]  TARC  [ **CCL17** ] .  Ref: 11876749 Clin Exp Immunol, 2002
- These facts suggest possible involvement of two  **CCR4**  specific ligandsi.e., thymus and activation regulated chemokine  [ **CCL17** ]  TARC  [ **CCL17** ]  and macrophage derived chemokine MDC, in the pathogenesis of bronchial asthma via recruitment of TH2 cells to inflammatory sites.  Ref: 11929424 Allergy, 2002
- To find out whether this mechanism might also be operative in CD30 malignant lymphomas other than HD, TARC  [ **CCL17** ]  and  **CCR4**  expression was investigated by immunohistochemistry on paraffin and frozen tissue sections of 39 nodal CD30 anaplastic large cell lymphomas ALCL, including 27 ALK negative and 12 ALK positive ALCL, 25 primary cutaneous CD30 ALCL, including 11 patients with lymphomatoid papulosis, and 31 cases of HD.  Ref: 12181269 Mod Pathol, 2002
- Supernatants from malignant cells cultured in the presence of sCD40L promote the migration of activated T cells that express  **CCR4** , the common specific receptor for MDC and TARC  [ **CCL17** ] .  Ref: 11468146 Blood, 2001
- Demonstration of TARC  [ **CCL17** ]  and  **CCR4**  mRNA expression and distribution using in situ RT PCR in the lesional skin of atopic dermatitis.  Ref: 12598706 J Dermatol, 2003
- Thymus and activation regulated chemokine  [ **CCL17** ]  TARC  [ **CCL17** ]   **CCL17**  and its receptor, CC chemokine receptor 4  [ **CCR4** ]   **CCR4** , have been proven to be involved in a number of allergic diseases, especially atopic dermatitis AD.  Ref: 12598706 J Dermatol, 2003
- Among  **CCR4**  ligands, skin vessels endothelial cells present C C chemokine ligand CCL 17  [ **CCL17** ]  but not CCL22 consistent with  **CCL17**  involvement in T lymphocyte arrest on endothelial cells.  Ref: 12193695 J Immunol, 2002
- The purpose of this study was to examine the expression and distribution of TARC  [ **CCL17** ]  and  **CCR4**  mRNAs in samples of AD n=15, acute lesions 8, chronic lesions 7 and normal skin n=6.  Ref: 12598706 J Dermatol, 2003
- TARC  [ **CCL17** ]  and  **CCR4**  may be involved in the pathogenesis of MF.  Ref: 12522366 J Am Acad Dermatol, 2003
- This was associated with the expression of their agonistic receptor CXCR3, while mRNA for TARC  [ **CCL17** ]  and MDC, and their receptor  **CCR4**  were not detected.  Ref: 11912011 J Dermatol Sci, 2002
- TARC  [ **CCL17** ]  positive cells and  **CCR4**  positive cells were rarely encountered in all cases.  Ref: 12688353 Leuk Lymphoma, 2003
- The  **CCR4**  TARC  [ **CCL17** ]  receptor positive lymphocytes, surrounding H& ; RS cells, were minority and the surrounding lymphocytes were not different between TARC  [ **CCL17** ]  positive and negative cases.  Ref: 11920617 Int J Cancer, 2002
- Differential expression of thymus and activation regulated chemokine  [ **CCL17** ]  and its receptor  **CCR4**  in nodal and cutaneous anaplastic large cell lymphomas and Hodgkin s disease.  Ref: 12181269 Mod Pathol, 2002
- Apart from three cases of HD showing 10 to 15%  **CCR4**  positive lymphocytes surrounding TARC  [ **CCL17** ]  positive H RS cells,  **CCR4**  positive reactive T cells were few < ; 5% in all other cases studied.  Ref: 12181269 Mod Pathol, 2002
- At 4 and 8 weeks after reconstitution of Rag 2 mice the mRNA levels of IP 10, MCP 1, MDC, MIG, TARC  [ **CCL17** ] , RANTES,  **CCR4**  and CCR5 were significantly increased prior to the appearance of macroscopic lesions.  Ref: 11465103 Eur J Immunol, 2001
- There was a significant correlation between the number of  **CCR4**  CD4 T cells and the levels of TARC  [ **CCL17** ] , MDC, and IL 5 in BALF of patients with EP.  Ref: 12757454 Allergy, 2003
- We examined the distribution of  **CCR4**  expressing CD4 and CD8 T cells in human decidua at early pregnancy, and localized TARC  [ **CCL17** ]  in the decidual tissue and chorionic tissue.  Ref: 12322891 Am J Reprod Immunol, 2002
- Our results demonstrate a differential expression of TARC  [ **CCL17** ]  and  **CCR4**  in different types of CD30 malignant lymphomas.  Ref: 12181269 Mod Pathol, 2002
- Moreover, we examined TARC  [ **CCL17** ] ,  **CCR4**  and CXC chemokine receptor 3 CXCR3 expression in the lesional skin of patients with BP by immunohistochemical procedures.  Ref: 12588369 Br J Dermatol, 2003
- Because in vitro studies showed that  **CCR4**  expression is a marker for lymphocytes bearing a T helper 2 Th2 phenotype, it was suggested that expression of TARC  [ **CCL17** ]  is a new immune escape mechanism in HD.  Ref: 12181269 Mod Pathol, 2002
- Importantly, mature dendritic cells preferentially attract Treg cells among circulating CD4 T cells, by secretion of  **CCR4**  ligands  **CCL17**  and CCL22.  Ref: 11560999 J Exp Med, 2001
- Furthermore, we detected strong signals for  **CCR4** , TARC  [ **CCL17** ] , and MDC in ATL skin lesions by RT PCR.  Ref: 11861261 Blood, 2002
- METHODS We investigated the expression of TARC  [ **CCL17** ] ,  **CCR4** , and CXC chemokine receptor 3 in patients with each stage of MF by immunohistochemistry.  Ref: 12522366 J Am Acad Dermatol, 2003
- Accumulation of  **CCR4**  expressing CD4 T cells and high concentration of its ligands TARC  [ **CCL17** ]  and MDC in bronchoalveolar lavage fluid of patients with eosinophilic pneumonia.  Ref: 12757454 Allergy, 2003
- and 4 an efficient migration of fresh ATL cells to the  **CCR4**  ligands, TARC  [ **CCL17** ]   **CCL17**  and MDC CCL22, in chemotaxis assays.  Ref: 11861261 Blood, 2002
- First, mRNA levels encoding the chemokines MIG, RANTES, lymphotactin, MIP 3alpha, TCA 3, TARC  [ **CCL17** ] , MIP 3beta, LIX, MCP 1 and MIP 1beta and the receptors  **CCR4** , CCR6 and CCR2 were significantly increased in chronically inflamed colons of IL 10 mice when compared with wildtype mice.  Ref: 11465103 Eur J Immunol, 2001
